# Dark Web Data Classification Using Neural Network

**DOI:** 10.1155/2022/8393318

**Published:** 2022-03-28

**Authors:** Anand Singh Rajawat, Pradeep Bedi, S. B. Goyal, Sandeep Kautish, Zhang Xihua, Hanan Aljuaid, Ali Wagdy Mohamed

**Affiliations:** ^1^School of Computer Science & Engineering, Sandip University, Nashik, Mahrashtra, India; ^2^Galgotias University, Greater Noida, Uttar Pradesh, India; ^3^City University, Petaling Jaya, Malaysia; ^4^LBEF Camus, Kathmandu, Nepal; ^5^Baicheng Normal University, Baicheng, China; ^6^Department of Computer Sciences, College of Computer and Information Sciences, Princess Nourah bint Abdulrahman University, P.O. Box 84428, Riyadh 11671, Saudi Arabia; ^7^Operations Research Department, Faculty of Graduate Studies for Statistical Research, Cairo University, Giza 12613, Egypt; ^8^Department of Mathematics and Actuarial Science, School of Science and Engineering, The American University in Cairo, New Cairo, Egypt

## Abstract

There are several issues associated with Dark Web Structural Patterns mining (including many redundant and irrelevant information), which increases the numerous types of cybercrime like illegal trade, forums, terrorist activity, and illegal online shopping. Understanding online criminal behavior is challenging because the data is available in a vast amount. To require an approach for learning the criminal behavior to check the recent request for improving the labeled data as a user profiling, Dark Web Structural Patterns mining in the case of multidimensional data sets gives uncertain results. Uncertain classification results cause a problem of not being able to predict user behavior. Since data of multidimensional nature has feature mixes, it has an adverse influence on classification. The data associated with Dark Web inundation has restricted us from giving the appropriate solution according to the need. In the research design, a Fusion NN (Neural network)-S^3^VM for Criminal Network activity prediction model is proposed based on the neural network; NN- S^3^VM can improve the prediction.

## 1. Introduction

Dark Web structural mining is measured as an essential portion of data analysis associated with cybersecurity. The primary purpose of structural data analysis is to extract relevant results to provide data consistency concerning the most prominent properties. This problem was initially proposed in the background of market basket analysis with instruction to discover frequent cybercrime activity collections that are accepted [1]. Subsequently, its prescribed classification, at initial, an extraordinary number of algorithms, has been designated [2]. Further, most of these algorithms are constructed on naive Bayes algorithm or Apriori-like methods creating a gradient of candidate items, sets, or patterns molded by several fusions of single items [3]. After the number of these solitary items to be collective, the pattern mining problem became a problematic assignment, and other effective approaches are compulsory. After that, the quantity of item sets in which a cylindrical container is produced is identical 2*n* − 1, so it develops enormously multifaceted through the increasing amount of singletons. The novel approaches are designed to explore dark web patterns created on antimonotone things as a pruning approach. It regulates that a sub-Dark Web Structural pattern of a recurrent Dark Web Structural pattern is similarly frequent, and either superpattern of an intermittent pattern determination is certainly not frequent. This pruning approach permits the search space to be complete after a pattern is noticeable as infrequent; then no novel pattern requirements are produced. The enormous volumes of data in numerous request fields have initiated a reduction in current approaches' performance. Traditional Dark Web Structural pattern mining algorithms are not appropriate for classifying data accurately, giving two significant problems to be resolved:

- Computational complexity
- Main memory necessities

Big data is the appearance of its gage, diversity, and velocity. Dark web Data streams remain typically incredible in velocity and real-time. Consequently, profligate and incessant active time processing is required to illustrate dark web big data's real significance. In the field of illegal trade, forums, terrorist activity, online shopping, websites have a massive quantity of data that has been compiled together in the existing criminal systems. The presence and request of the online news have complete traffic data additional to similar big data. As a consequence, time and investment costs are reduced. The existing SVM and neural network algorithm based prediction model has the benefits of high accuracy and extraordinary simplification, resolves the problems of minor illustrations and high dimensional illustrations, and successfully forecasts classified cybercriminal activity according to requirement [4]. Prediction model is innovative to classify the results online and progress its accuracy. The neural network model can correspondingly be used to predict movement flow. It mostly accepts matrix assessment to improve the neural network algorithm. Its accuracy is improved than customary neural network representations. Our proposed approach is based on the concept of the Semisupervised Algorithm and Constructive Semisupervised Classification (CSSL) Algorithm. The paper is organized in the following format.  Section 2 represents the related work; Section 3 shows the challenges: dark web structural patterns data classification; Section 4 presentsdark web structural patterns mining data; Section 5 describes SVM web structure for data classification; Section 6: presents optimization in support vector machines; Section 7 presents proposed methodology; Section 8 provides discussion; Section 9 summarizes conclusion and future work.

## 2. Related Work

The performance of [5] the model for deep reinforcement learning (DRL) shows an appreciable result when compared with conventional supervised techniques of ML (Machine learning) like GBM (gradient boosting machine technique) for criminal network analysis (CNA). The techniques used here contain CCND (Corrupted Criminal Network Dataset) to reconstruct models for link prediction training by using the features of DRL Techniques (deep reinforcement learning). The techniques given by [6] presented the approach of analyzing dark web data managing processes using discrete principles and practices designed for respective stages. [2] presented the approach of identifying core site links using the examination of hyperlinks SNA (Social Network Analysis). The density value of 0.132 is achieved and shows a significant section of core links and sites. The Olivier Chapelle et al., 2008 Research focuses on S3VM (Semisupervised Support Vector Machines) to further study the literature review and available techniques to enhance the S3VM algorithm. The comparative study and literary analysis are done by [7] to present deep learning models' performance in solving the complex network issues used to predict network values with acceptable result parameters. The image-based network transformation of the Matrix (adjacency) approach is given by [8] using DGM (Deep Generative Modeling techniques) for understanding the hierarchical parameters and features. The reviewed literature is discussed by [9] the systematic construction of identified issues chosen network values of datasets to analyze link prediction features. The machine learning ensemble approach was given [10] for identifying the interfacing issues using NN and DT (decision-Trees) algorithms. The Bayes algorithms and maxim entropy techniques were implemented [11]. For analyzing the data required for training purposes, the cost-based semisupervised approach (CS4VM) [12] approximates SVM supervised values of cost used in link prediction modeling for the network [13]. Based on the findings of recent studies, it appears to be an effective method of discovering whether or not the program has been pirated in any significant way. Reference [14] presents the Haar wavelet collocation method (HWCM) for solving linear and nonlinear Schrödinger equations numerically. Through the use of linearization, the nonlinear term in the model equation is made linear [15]. For PDEs related to the framework of the so-called inverse problem, a Haar wavelet collocation method (HWCM) is developed [16]. Methods for solving linear PDEs with an unknown source of heat and an identifiable solution are described in this paper in two separate versions. Two different multiresolution approaches based on Haar wavelets are shown here for numerically solving the Schrödinger equations in two dimensions. The linear and nonlinear model equations are put into consideration [17]. In order to address the inverse problem of ambiguous source control parameters, a hybrid Haar wavelet collocation method (HWCM) has been proposed. These problems are difficult to answer numerically because they have nonlinear components and uncertain control parameter sources. Nonlinear hyperbolic Schroedinger equations (NHSEs) can be quantitatively solved using a Haar wavelet collocation technique (HWCM). There are two ways to estimate the time derivative in the governing equations: by utilizing finite Haar series or by employing space-derivatives. An investigation into educational institutions' understanding of piracy is the goal of this study. Piracy and awareness of pirated software can be determined through this investigation, which can ultimately benefit academics.

## 3. Challenges: Dark Web Structural Patterns Data Classification

- Instinctive learning and dynamic behavior
- Runtime active environments for feature selection
- Estimating a system for normal behavior and contribute to the personalized outcome.

Predictable dark web structural patterns mining algorithms for crime activity information extraction and classification using neural network algorithm (just like a backpropagation neural network) are not appropriate for colossal data platforms (dynamic change and uncertain) based on the Mapper Reduce model [18]. Furthermore, when the data measure is enormous (typically happening the size of gigabytes), this algorithm works very slow and cannot generate output completely. This research examines machine learning and a deep learning algorithm based on web usage mining, fusion level deep learning-based on a backpropagation neural network using binary classification. It proposes a Fusion deep learning model based on map reduction applied on usage data sets to modify the performance in terms of accuracy over huge-scale web usage data. This model is called NN-S^3^VM (neural network and semisupervised support vector machine) [19].

To apply the fusion of two algorithms, SVM and semisupervised, the two algorithms' association is done through collaborative learning. We can devise a novel tritraining or cotraining algorithm by expanding different expectations. The vast number of the numerous techniques and algorithms of dark web structure data classification useful to developing the criminal data classifiers are currently identified. Such techniques and algorithms are planned, for example, to develop the linear SVM, SVMG-RBF, BPNN, S^3^VM, the decision trees, the significant rules of classification, deep learning, and more. The classical SVM model is problematic to evaluate huge major useful problems. Parallel SVM can improve the execution speed significantly. The high classification value was proposed for dark web activity in specific, the SVM algorithm (Support Vector Machine Algorithm), and the SVMG-RBF show BPNN, S^3^VM.

Though, presently no data classifier can completely specify or resolve local minima problems. Existing tools cannot solve classification with high-quality dark web structure data since tools have limitations for a particular dark web structure data. Consequently, the decision on the fusion of the SVM and SSL (Semisupervised learning) for classification of the elegant mix dataset (illegal trade, forums, terrorist activity, online shopping dark web Criminal Network) was accepted [20]. Collaborative filtering techniques are frequently used to resolve such problems based on dark web user-to-user correspondence or rely on matrix factorization methods to construct hidden factor vectors for every user. To propose a novel model of NN-S^3^VM, a semisupervised support vector machine based on a map-reducing model for data classification using the fusion-based deep learning model with a backpropagation neural network is proposed.

The existing fusion dark data classification includes much redundant and irrelevant information. The feature dimension reduction and information extraction can be worked on to eradicate inappropriate and redundant information successfully. To enhance the machine learning algorithm's efficiency, expand the accuracy of multidimensional dark web structure data classifications and predicting outcomes and increase learning capability.

## 4. Dark Web Structural Patterns Mining Data

The pattern matching techniques for dark web is related to textual data in for of logs (records). However, the data can be classified on different techniques for data mining.

### 4.1. Dark Web Click Stream Data

Using our approach can determine cybercriminal interest and their accomplishments in different problems like illegal trade, forums, terrorist activity, inspecting, and more. It produces analyzers of the patterns and intelligently visualizes the customers' analogous type of products.

### 4.2. News and Sentiment Analysis

Dark web News and Dark web Sentiment data is unlabeled dark web that characterizes opinions, emotions, and attitudes defined in sources such as blogs, social media posts, online newspapers, online product reviews, and consumer support communications. Dissimilar companies and organizations use social media analysis to appreciate how the public feels about some issue at a specific moment in time and similarly track down how those sentiments modify over time.

### 4.3. Dark Web Trending Volume

Now voluminous dark data is converted to the number of jobs, and jobs can be quickly processed using the proposed framework. Trending Volume is a big concern currently. Day by day, it has been accumulative at a considerably higher rate in the establishments and social media sites, and more.

### 4.4. Dark Web Predictive Analytics

This analytics is the Dark web big point in our research. It provides predictive scores to the organizations to support in creating smart decisions and dark website behavior to proliferate customer responses in business, adaptations, and consultations.

### 4.5. Dark Web Text Analytics

This analytics is the process for originating the high, prominent information from the raw data, such as unstructured data and forecasting and predicting the analysis.

### 4.6. Dark Web Social Media Mining

Through HADOOP, mine Facebook and other social media discussions for people's sentiment data and use it to produce targeted real-time decisions.

## 5. SVM Web Structure for Data Classification

Primarily, SVMs were established with the investigation abilities and volume control of machine learning and to resolve overfitting complications in high-dimensional feature spaces preowned formalization. SVMs can make decisions by reducing classification errors, subsequently minimalizing so-called operational risks. SVMs can reduce the misclassification problem with the help of maximum probability techniques (MPT). Support vector machine can directly specify the distribution of training sets. Presently, SVMs are associated with the nonparametric supervised classification technique. It has been accepted as the domain of machine learning and pattern recognition. The best technique for dark web structure data classification is a support vector machine used for hyperplane optimization [21]. The main concept is to divide it into several classes. SVM uses Maximum Margin Classifier (MMC) for resolving the particle problem. Vapnik introduced a Support Vector Machine (SVM). To apply the classification, the training dark web stricture data set is represented as follows:

(1) $$\begin{array}{c} \text{DS}=\left\{X1,\ldots ,X_{p}\right\}, \end{array}$$

where *x*_*i*_ is specific vector labels will be represented as follows:

(2) $$\begin{array}{c} \left\{X_{1}^{\prime },\ldots ,X_{q}^{\prime }\right\}. \end{array}$$

SVM classifies the dark web stricture data into two classes: training class and testing class. The number of hyperplanes can be associated with the two classes. SVM classifier chooses the hyperplane to compute the minimum distance between the cluster. Distance is computed through margin. One side hyperplane can be represented as *a* − 1 labeled. Another side hyperplane can be represented as *a* + 1. It represents that support vector training information traveling between neighbor and SVM.

*Classification*: *γi* is a Functional margin and represents the number of an instance (*x*_*i*_, *x*_*i*_′). It describes the hyper plan in the opposite direction to a hyper-plane (*w*, *b*), written as follows:

(3) $$\begin{array}{c} \gamma i=x_{i}\left(\langle w,x_{i}\rangle +b\right). \end{array}$$

To show the positive margin as a *γi* > 0 and recommendation margin of (*x*_*i*_, *x*_*i*_′). The data point is separated and labeled among the hyperplanes. Between hyperplanes that distinct the labeled points, a unique point provide a more margin between the data point. Using the support vector machine, discover the highest margin between the hyperplanes. However, the functional margin has been computed to represent *w* and *b*. Therefore, it is represented as a geometric margin *γ*g, The full margin is utilized to create the rules-based margin.

## 6. Optimization in Support Vector Machines

SVM has a problem with optimization. Several algorithms work to solve the optimization problem.

### 6.1. Semisupervised Algorithm for Web Usage Mining

SSL algorithm is a machine learning algorithm that is used for the dark web stricture data classification. Raw dark web structure data set or mixed data set can be categorized as a labeled data set and unlabeled data set. Several machine learning algorithms work for the classification of unlabeled data set. The SSL algorithm is very powerful for information extraction in the unlabeled dataset.

It is easy to classify the low amount of data using SSL, but current scenario data are generated in huge or significant quantities and dynamically; thus, an efficient algorithm for training and uncertain dark web stricture dataset is required. The training and testing using a semisupervised algorithm can classify unlabeled data set into the labeled dataset.

Training and testing are performed using a semisupervised support vector, which simplifies the instance according to the given training set. The fusion-based technique is used for performing the training with transitive learning. It is a simple way to classify the unlabeled sample. To discover the knowledge in the unlabeled data, the margin is checked between labeled data. Our proposed model is used for analysis and finding out the improvement in SVM.

### 6.2. Microclustering Algorithm (MCA) for Large Dark Web Stricture Data

In the order of ranking microclustering computation, it has the following peculiarities. It conceptualizes a microidentically compact grouping tree, proclaimed to be a CF (Clustering Feature) tree. The information set going through the validation process indicates the inactive measure of resources by regular small measuring and innovatively accessing multidimensional information points in identically compact groups.

After the same, the specific checking of the numbers no longer allows backing out. Common inaccuracies might be likely to take place, believing in the sequence of numbers entered. The CF tree takes possession of the studied numerical data's prominent delivery patterns and advises required information for SVM to gently accept it. It manages noise or outliers skillfully as a copy of the clustering [22]. SVM coordinates a line or a hyperplane among binary sets of data for classification. The data are taken data *X* that comes under a different aspect of the hyperplane.

(XT•*W*–b) > 0, arc labeled as +1 and which lessens on the extra side.

(XT•*W*–b) < 0, arc labeled as −1.

### 6.3. Recursive Map Reduce Model for SVM

Gaussian kernel (Kernel function) and its numerous selections of the parameters should be examined to discover the preeminent SVM. Applying and processing massive dark web structure data set for training and testing (Algorithm 1).

**Figure 1:**
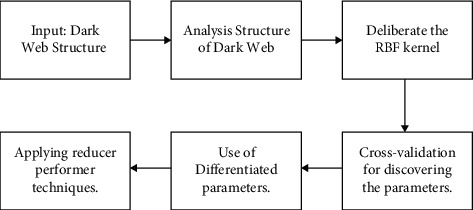
Flowchart of the proposed algorithm.

The flowchart of the algorithm that has been proposed is depicted in Figure 1.

### 6.4. Constructive Semisupervised Classification (CSSL) Algorithm

This section discusses details required to implement constructive semisupervised learning (CSSL) based on the classification [23] of system spending perception of geometrical growth. To explain the algorithm for classification, the semisupervised learning [24] Algorithm is essentially constructed over multilevel geometrical. The know neurons can be extracted when it is represented by hyperspheres presentation, so they are given a learning task [9] using hyperspheres performed by the geometrical growth. CSSL Algorithm has a particular advantage over previous learning algorithm for Backpropagation Neural Network (BPNN) [25]:

- Design and Implementation of CSSL algorithm that keeps the simplification ability and compares existing learning algorithms.
- CSSL algorithm does not need previous vertex training, which creates its additional simplicity.
- CSSL algorithm required a short time for learning. It gets it regularly to permit a vertex to a conceived neuron or by managing to which specific area it belongs to or not making use of the loaded sum of the single specific arrangement of a vertex.

Dark Web Structural Patterns mined using neural network-S^3^VM for Criminal Network are research domain of computer technology information whose development is done by the improvements in data assessment research, tracking, and escalation in the cybercrime activity. The dark web criminal market needs activity tracking approaches that might help extract appreciated information from massive data stores.

## 7. Proposed Methodology

Our proposed technique is to improve the performance of SVM [26] and boost the SVM speed and Radial Basis Function (RBF) kernel function using the machine learning technique [27]. Machine learning is the perception of classification, learning, and analysis [28]. In this concept, labeled and unlabeled classes of data together can be evaluated. Labeled dark web data can be created using unlabeled dark web structure data, and unlabeled web structure data is taken from web usage data. Several dissimilar [29] types of approaches are available for labeled data processing, but there is an insufficient quantity of unstructured data exploration methods and their accurate analysis. The presented work is keen on discovering a resourceful and accurate framework by which the unlabeled (mixed data set) data and their classification can be analyzed uniquely. To find such a procedure, various techniques are evaluated to determine the best technique with maximum capability.

The experimental results of our proposed neural network-S^3^VM model are obtained by evaluating using SVM. Our proposed model for the classifier, the performance of the existing classification technique is improved. The proposed technique is based on the deep learning model's [30] fusion, binary neural network classifier, and expanding the backpropagation neural network (BPNN). In a significant step, labeled data is used with binary classification. The probability of every dataset in the classification class is positioned. Afterward, the computed probability is converted into weights. These weights are disseminated in both definite classes and neural network training. Throughout the classification, testing data is again studied for a similar progression, and weights are readjusted for essential labels of the dataset. The output of this testing is used to predict the performance of the scheme [31]. We present a simplified algorithm through neural network-S^3^VM. The outputs of our algorithm have been tested through experimental analysis.

Our data classification technique is an improvement over the previous distributed or parallel works in two ways. One new training neural network-S^3^VM algorithm and deep learning backpropagation neural network classification to obtain classifier function. Secondly, applying more feature engineering methods increases the overall accuracy of the system. Applying our approach reduces the web application problem for classifying and improves big data accuracy; Big data is quite common nowadays. The results of this research are essential for the training of datasets for neural network-S3VM [32] algorithm-based classification problems.

S^3^VM for information recommendation according to the user interest, applying the hybrid technique to use minimum classified the data web structure data in a raw data set, applying training and testing to generate the large training sample and less testing sample. Using our proposed model, conversion of large unlabeled instances into labeled instances depends [33] on the fusion level.

The most significant task for fusion-based future selection algorithms can improve accuracy and proficiency. We have illustrated simulation on real-time data set, generated effective results, and improved the efficiency of the map-reduced model.

### 7.1. Neural Network Approach

The study of this network will aid us in gaining insight into the fundamental reasons behind the complex Deep Learning models [34] that we are currently investigating. The Multilayer Perceptron is an example of a type of neural network that is widely used in simple regression scenarios and is a type of neural network. When it comes to pattern analysis, MLPs are not particularly well suited, especially for sequential and multidimensional data, according to the literature. A multilayer perceptron must have a “large” number of parameters in order to successfully handle multidimensional [35] data as a result of these considerations. The fact that they can cope with both sequential and random input is one of the reasons why RNNs are so commonly utilized. As a result of its patterns, the network [36] is able to identify dependencies on earlier data, which is extremely important when making predictions. When it comes to extracting resource maps from data sources such as images and videos, CNNs are particularly adept. These maps may subsequently be used for classification and segmentation [37], among other things. The term convolutional neural network (CNN) refers to a neural network that receives sequential input data and is constructed using a convolutional neural network (CNN) in the form of Conv1D/1D. Using a combination of MLPs, CNNs, and RNNs in most Deep Learning models, it is possible to maximize the effectiveness of each of the three types of deep learning models. It is not possible for LP, CNN, and RNN to complete all of their objectives. A substantial percentage of its overall effectiveness can be attributed to the fact that it was able to identify its purpose [38] and choose the most appropriate parameters, such as the Loss function, the Optimizer, and the Regularizer, early on. Furthermore, we have access to information that has been obtained outside of the training environment. The Regularizer is used to ensure that the trained model generalizes to fresh data, which is essential in machine learning.

### 7.2. Evaluation of Our Proposed Framework

We improve upon existing SVM algorithms by including new features. To improve the quality of working of SVM algorithms, To develop a semisupervised algorithm, the semisupervised algorithm can help us determine the navigation ratio from positive to negative values properly [39]. This semisupervised algorithm does not contain any active learning, so it cannot correctly select the best samples. In this semisupervised algorithm, when the size of the training sample increases, the speed gets slow. A novel framework using algorithms for improving the accuracy of existing SVM is proposed. Semisupervised learning algorithm is proposed through S^3^VM in multilevel classification based *n* neural network algorithm. Traditional approaches have limited efficiency [40].

To perform the simulation, our experiment uses a fusion level algorithm implemented on Java and Python language using the HADOOP framework and is based on a map reducer model. Our working model is useable and applicable to compute the complexity, improve accuracy, and minimize the error rate. This model is estimated compared with a traditional classifier, specifically SVM (support vector machine). SVMG-RBF shows BPNN, S^3^VM. Our method has the following rewards: primarily, our learning strategy classified the labeled data set at a very high accuracy level, generated the multidimension profile, and classified the confidential information [41] that was not classified. This research work proposes raw data set classification through NN- S^3^VM and used Map-reduce based model in HADOOP. To increase the accuracy and reduce error rate, memory consumption, time consumption by feature space regression, and parameter alteration, a map reducer is used for the proliferation of execution speed. Previous researches enhanced the execution speed of S^3^VM through linear kernel function [42]. The proposed framework boosted the speed of S^3^VM with RBF kernel function using the Map reducer model. Improved S^3^VM (NN-S^3^VM used Map-reduce based) has been compared [43] with the simple SVM, TSVM, NN- S^3^VM.

#### 7.2.1. Preprocessing of Dark Web Structural Patterns Information

Preprocessing performs several tasks. The first task is applying the mixed data set for cleaning the data; the second task performs the session identification according to [44] user usage data perspective.

#### 7.2.2. Discovery of Dark Web Pattern

Data produced by preprocessing phase finds the required pattern, then applies the number of data mining algorithms based on machine learning sequential pattern technique, semisupervised learning algorithm [45], classification, K-means clustering, and to discover valuable information.

#### 7.2.3. Dark Web Pattern Analysis

This is the most impudent task in data mining. Patterns are analyzed for dark web customer behavior prediction [46]. Many algorithms and tools have been developed to analyze the pattern, such as deep learning algorithms [47] and supervised algorithms.

**Figure 2:**
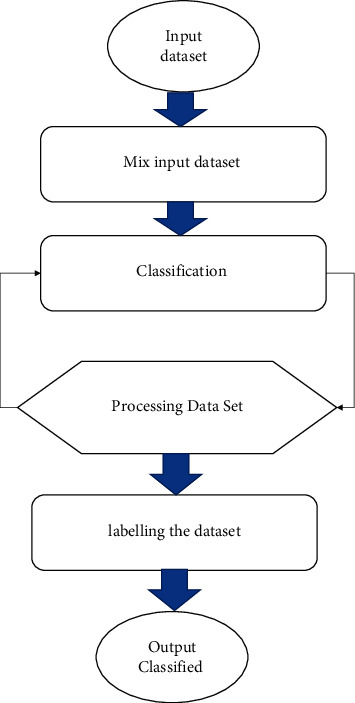
Data Preprocessing stage.

The data preprocessing stage of the process is depicted in Figure 2 of this document. We selected the dark web criminal Network dataset (Scraping data). The data set has information about the dark web node, edge, the link between the paired node. We examine this behavior, and we calculate the speedup metric defined in the equation below. The speedup can be amounted to how the accumulative number of nodes benefit the performance. Figure 2 illustrates that the presentation of 10 nodes is saturated after the data size is around 2000 MB, while the speedup of 30 and 40 nodes designates excellent realizable performance and parallelism. The solitary abnormality originates in the speedup of 50 nodes; subsequently, it is lower than those of 30 and 40 nodes. This might be owing to the permeation of bandwidth shared amongst nodes in notable change. Response time = *R*, Sequential = *SQ*, parallel = *p*, Speed up = *S*

(4) $$\begin{array}{c} \text{Speed up}\left(S\right)=\frac{R\left(SQ\right)}{R\left(P\right)}. \end{array}$$

**Figure 3:**
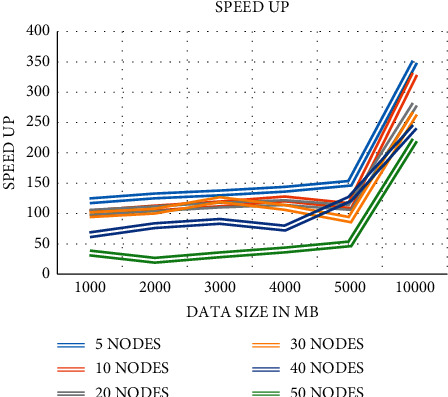
Speedup performance of the proposed model.

Speedup is illustrated in Figure 3 as a measure of the performance of the proposed model.

An additional metric that needs to be measured is efficiency. The efficiency designates in what way the scheme achieves after adding additional nodes. It is distinct as the number of nodes separates the speed up. It is effective as soon as the number of nodes is amplified. Perceptibly, the efficiency diminishes when the quantity of nodes is improved [48] for complete dark web dataset sizes; then, it proliferates once the dataset size is improved.

The throughput is the quantity of comprehensive managed data per unit of time. In this research, the quantity is the size of the handled data separated by the response time as defined below. Throughput = *T*, *R* = response time, data processing size (DPS)

(5) $$\begin{array}{c} T=\frac{DPS}{R}. \end{array}$$

**Figure 4:**
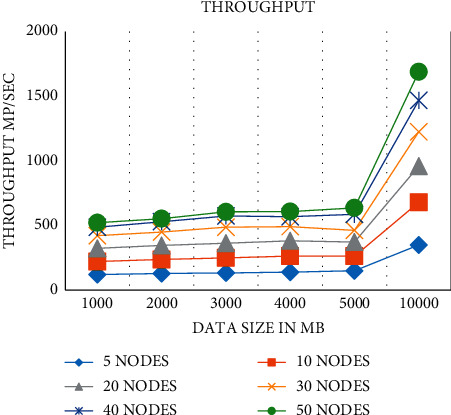
Average throughput.

Figure 2 illustrates the throughput in MB per second when the amount of nodes is improved. They illustrate comparable leanings through the consequences of the speedup. The throughput is improved with the number of node growths and the data size intensifications in the additional difference of estimation.  Figure 4 shows a representation of the average throughput.

We proposed a model for investigating and classification of dark web structure usage data into the standard. The typical dark web structure usage data designate consistent behavior of dark web structure usage originating in maximum establishments, and they are calculated for more than 90%. Since the volumes of usage data reserved in storage are frequently massive, filtering them at the primary period of usage data supervision would save enormous storage.

We classify usage data, and we calculate the threshold rates of different file groups and file categories; subsequently, they have distinct features. The usage data would designate possible uncharacteristic behavior of dark web structure usage. We similarly quantify the performance of the usage data classification using a HADOOP system, subsequently relating its parallel contrivance, which would benefit the performance of usage data processing a lot. The investigational consequences have exposed a respectable development of performance enhancement. Receiver operating characteristic (ROC) is the graphical representation for a binary classifier system.  Table 1 contains a comprehensive description of the software used by the modules.

### 7.3. Parameters of Evaluation Used to Measure Performance

To assess the efficacy of the various algorithm, we used True Positive Rate (TPR), False Positive Rate (FPR), Precision, and Precision. The following definitions are TPR, FPR, Accuracy, and Consistency.

(6) $$\begin{array}{c} \text{Accuracy}=\frac{\text{TN}+\text{TP}}{\text{TP}+\text{TN}+\text{FN}+\text{FP}},\\ \text{Precision}=\frac{\text{TP}}{\text{TP}+\text{FP}},\\ \text{Recall}=\frac{\text{TP}}{\text{TP}+\text{FN}},\\ \text{F}1\text{Score}=2*\frac{\text{Precision}*\text{Recall}}{\text{Precision}+\text{Recall}}. \end{array}$$

Detailed results of the simulation are presented in Table 2.

Table 3 shows the accuracy and effectiveness of the models (SVM, TSVM, NN-S3VM).

**Figure 5:**
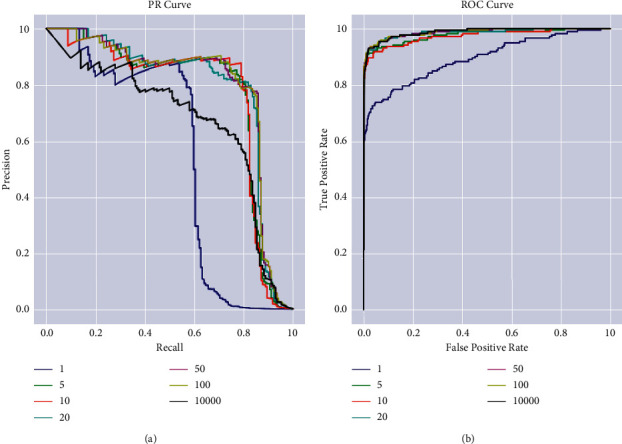
(a) PR curve. (b) ROC curve.

In Figures 5(a) and 5(b), PR curve and ROC curve describe the working of the proposed modified support vector machine algorithm [25, 26]. The two graphs were ranges between 0.0 and 1.0 on the *y*-axis and *x*-axis. The first graph is plotted between precision and Recall (PR curve) and the other graph is plotted between true positive rate and false positive rate.

## 8. Discussion

Using dark web patterns, antimonotone patterns can be pruned. The frequency of determining an intermittent Dark Web Structural Pattern and the superpattern of a recurring Dark Web Structural Pattern. This type of pruning allows the search space to be completed after a pattern is identified as unusual. Massive volumes of data in numerous request fields have slowed current approaches. Traditional Dark Web structural pattern mining approaches have two fundamental flaws. Requirements for main memory and computational Big data's magnitude, diversity, and speed make it so. Shadows Data streams remain extraordinarily rapid and real-time. Thus, excessive and continuous active time processing is necessary to demonstrate the actual value of dark web big data. Existing criminal systems collect a great quantity of data on illegal trade, forums, terrorism, Internet purchases, etc. The online presence and demand for news have complete traffic figures. As a result, time and money are saved. The SVM and neural network algorithm based prediction model is recommended because of its high accuracy, incredible simplification, and capacity to handle tiny and high dimensional pictures. Our technique for forecasting outcomes is cutting-edge. The same neural network model can forecast movement flow. The widely acknowledged matrix assessment helps improve the neural network algorithm.

## 9. Conclusion and Future Work

Dark Web Structural Patterns mining using neural network-S^3^VM for Criminal Network has a significant role in different circumstances. It has been used in different domains, and it is an increasing range of knowledge. Though the types of data are assorted, these data sets are contingent on the application's nature. With different situations of data, the method of classification varies. We classify information from the dark Web Structural Patterns dataset varieties. It is easy to gain the parsed data to additional levels where it is not distinct over time. We Select the Data set with multiple dark web domains. In the research design, a Fusion NN (Neural network)-S^3^VM for Criminal Network activity prediction model is proposed based on neural network, NN- S^3^VM can improve the prediction. We can try to implement a real-time scenario for criminal activity tracking using an artificial neural network in the future.

## Figures and Tables

**Algorithm 1:**
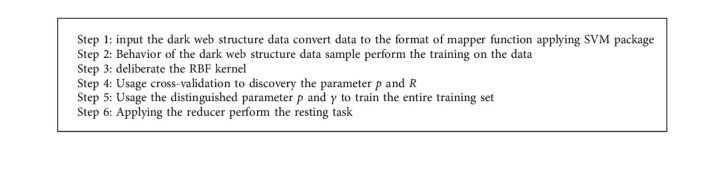
Map-reduce model for SVM program procedure.

**Table 1:** Software description of the modules.

Module	Software
*Data preprocessing*	*Hadoop*
Scraping tool	Dark search, TorBot, Fresh Onions, onioff, TorCrawl
SVM, TSVM	Scikit-learn

**Table 2:** Simulation results.

Experiment	Precision%	Recall%	F1 score %	Percentage of dark web link prediction
SVM,	72.02	13.02	20.34	32.31
TSVM,	75.23	13.91	20.23	39.02
Multilayer perceptrons (MLPs)	76.33	15.23	25.43	45.32
NN- S^3^VM	79.43	23.03	35.21	61.02

**Table 3:** Performance of models (SVM, TSVM, NN-S3VM).

Database	SVM			TSVM			NN- S^3^VM		
	Accuracy (%)	MCC	ROC area	Accuracy (%)	MCC	ROC area	Accuracy (%)	MCC	ROC area
CIRA-CIC-DoHBrw-2020	0.61	0.54	0.72	0.65	0.34	0.74	0.81	0.55	0.82
CSE-CIC-IDS2018 on AWS	0.63	0.28	0.82	0.64	0.31	0.83	0.84	0.34	0.83
Intrusion detection evaluation dataset (CIC-IDS2017)	0.64	0.30	0.92	0.64	0.25	0.93	0.84	0.29	0.94
Intrusion detection evaluation dataset (ISCXIDS2012)	0.71	0.50	0.91	0.76	0.49	0.92	0.87	0.13	0.95
DDoS evaluation dataset (CIC-DDoS2019)	0.74	0.30	0.92	0.78	0.29	0.94	0.91	0.13	0.96
Investigation of the android malware (CIC-InvesAndMal2019)	0.73	0.31	0.93	0.79	0.34	0.95	0.93	0.18	0.91
Android botnet dataset	0.71	0.51	0.94	0.81	0.41	0.95	0.94	0.46	0.97

## Data Availability

The data that support the findings of this study are available upon request from the corresponding author.
